# Avoidable workload of care for patients living with HIV infection in Abidjan, Côte d’Ivoire: A cross-sectional study

**DOI:** 10.1371/journal.pone.0202911

**Published:** 2018-08-24

**Authors:** Viet-Thi Tran, Mariam Mama Djima, Eugene Messou, Jocelyne Moisan, Jean-Pierre Grégoire, Didier K. Ekouevi

**Affiliations:** 1 Programme PAC-CI, Abidjan, Côte d’Ivoire; 2 METHODS Team, Centre de recherche en Epidémiologie et Statistiques Sorbonne Paris Cité (CRESS, UMR1153), Paris, France; 3 Institut Pasteur, Abidjan, Côte d’Ivoire; 4 Faculty of Pharmacy of Laval University, Québec, Canada; 5 Bordeaux Population Health (UMR1219), Bordeaux, France; Kurdistan University of Medical Sciences, ISLAMIC REPUBLIC OF IRAN

## Abstract

**Objective:**

People living with HIV infection (PLWHIV) in Sub-Saharan Africa cope with an increasing workload of care (doctor visits, lab tests, medication management, refills, etc.) in a context of poor health service organization. We aimed to describe the workload of care for PLWHIV in Sub-Saharan Africa and assess to what extent simple adjustments in care organization could reduce this workload of care.

**Methods:**

Adult PLWHIV under antiretroviral treatment for at least 1 year were recruited in three centers (two public, one private) in Abidjan, Côte d’Ivoire. Using methods inspired from sociology, we precisely described all health-related activities (HRAs) performed by patients, in 1 month, in terms of time, money and opportunity costs. Then, we assessed the theoretical avoidable workload of care if patients’ visits and tests had been grouped on the same days.

**Results:**

We enrolled 476 PLWHIV in the study. Patients devoted 6.7 hours (SD = 6.3), on average, in HRAs per month and spent 5% (SD = 11) of their monthly revenue, on average, on health activities. However, we found great inter-patient heterogeneity in the mixture of activities performed (managing medications; dietary recommendations; visits, tests, support groups; administrative tasks; etc.) and their time allocation, temporal dispersion and opportunity costs (personal, familial, social or professional costs). For 22% of patients, grouping activities on the same days could reduce both time and cost requirements by 20%.

**Conclusion:**

PLWHIV in Côte d’Ivoire have a heavy workload of care. Grouping visits and tests on the same days may be a simple and feasible way to reduce patients’ investment of time and money in their care.

## Context

With the roll out of antiretroviral therapy (ART), HIV has transitioned from an acute toward a chronic condition with life expectancies close to those from uninfected populations [1]. Despite this evolution, living with HIV now requires the adjustment of patients to incorporating a lifelong illness and its care into their identities, lives, daily routines, and interactions with others. People living with HIV infection (PLWHIV) have to cope with a heavy workload of care.

The workload of care is defined as the sum of all health-related activities (HRAs) imposed on patients’ lives, such as obtaining and attending appointments or exams, managing drugs, self-monitoring or lifestyle changes [2]. For PLWHIV in Sub-Saharan Africa, the workload of care is likely to be aggravated by 1) the aging of the population, who develop other chronic conditions not related to HIV, such as hypertension, diabetes or cancer, each requiring specific exams, treatments and follow-up [3–5]; and 2) the poor health service organization and delivery [6] in which patients often face fragmented care, long travels to health centers with high transportation costs [7] and hours of queuing to obtain treatment [8].

It is critical to take into account the “chronic” workload of care imposed on PLWHIV to achieve viral suppression and maintain it lifelong [9, 10]. According to the Cumulative Complexity Model, if the workload of care (i.e., the “objective” quantity of work imposed on patients by care) exceeds patients’ capacity, it may result in burden of treatment for patients (i.e., impact of care on patients’ wellbeing and quality of life) [6, 11, 12]. In the HIV literature, the balance between workload and burden has been described in the value/cost model. In this model, the workload of care and its associated burden can be considered as “costs” of healthcare. Whenever the “costs” of healthcare competes with other meaningful life demands such as patients’ social, family and professional lives, patients weigh the expected benefits of pursuing it against the burden of this care and may decide to intentionally not adhere to care [6, 12–16], which results in possible viral resistance to ART [17, 18], virological failure [19, 20], and increased mortality. To our knowledge, only a handful of studies have estimated PLWHIV’s workload of care, and these studies usually focused on selected aspects of this workload (e.g., distance, financial costs) while not considering it globally [7, 8, 21].

Objectives of the present study were to describe the workload of care of PLWHIV in a Sub-Saharan Africa setting and assess to what extent the workload of care of PLWHIV could be reduced by simple reorganization in visits’ schedule.

## Methods

The MOTUHS-BOT project was a cross-sectional study nested in the ANRS 12335 project, MOTUHS. MOTUHS was a large pharmaco-epidemiological study on drug treatments used by PLWHIV. In MOTUHS, consecutive HIV-1–infected adults in Côte d’Ivoire were enrolled in a cross-sectional study involving a survey about the medications they took regularly (including traditional medicines) and a viral load quantification. All participants from the three study sites of the MOTUHS study in Abidjan were invited to participate in MOTUHS-BOT. The study protocol for the MOTUHS-BOT study is available at on our institutional website [22].

### Participants and setting

Patients were consecutive HIV-1–infected people aged 18 years or older who received ART for at least 1 year and attended consultations in two public centers (the *Centre de Prise en Charge et de Formation* [CePReF] in Yopougon and the *Centre de suivi des donneurs de sang du Centre National de Transfusion Sanguine* [CNTS] in Treichville and one private center (the *Centre Intégré de Recherches Biocliniques* [CIRBA] in Treichville). These sites have more than 15 years’ experience in HIV care. All patients provided written informed consent before participating in the study. The study was approved by the National Ethics Committee from the Ministry of Health of Côte d’Ivoire. All methods were performed in accordance with the relevant guidelines and regulations.

## Assessment of patients’ workload of care

Three research associates collected information on participants’ workload of care during semi-structured interviews using a method inspired by the daily reconstruction method (DRM) [23]. The DRM is a hybrid approach that combines a time-use study with a technique for recovering affective experiences to precisely reconstruct all HRAs performed by patients in their context.

First, participants recalled all HRAs performed in the last 30 days during an unstructured discussion starting with the question: “Could you tell us about all the things you did in the last 30 days that were related to your health or care?” Investigators helped them by using a history calendar personalized with patient-reported landmarks (i.e., important life events that occurred in the time period such as birthdays, holidays, weddings, etc.) [24] and specific cues to improve the completeness of answers (e.g., “Did you have to make an appointment, attend visits or tests, visit traditional care providers, refill medications, retrieve results from tests/exams?”).

Then, for each identified HRA, participants answered a series of structured questions. First, they clarified the nature of the activity as medication management, physical exercise, dietary changes, appointment organization, visits to healthcare providers, exams or tests, visits to traditional practitioners, visits to the pharmacy, organization of drugs at home, health-related shopping, attendance at support or educational groups, or health-related administrative tasks. Second, they assessed the time spent on the activity by specifying the time when the activity began and ended, including transportation and waiting times. Third, they reported the direct financial costs involved (e.g., money spent on transportation, doctor visits or medications). Finally, they reported the opportunity costs of the HRA. Opportunity costs were defined as activities patients would have performed if they had not performed the HRA. They were investigated by the following question: “If you did not have to go to the doctor on that day, what would you have spent your time on?” Answers were classified with a list used in similar surveys [23]: working, engaging in social activities, housework, relaxing, eating, napping, watching television, shopping or enjoying leisure time.

### Other data collected

Besides information on participants’ workload of care, we obtained information on their self-reported adherence behaviors for ART using the questionnaire by Sidorkiewicz et al. [25] This 5-item tool describes patients’ adherence behaviors for each drug taken on a scale from 1 (absence of schedule errors and no omissions or drug holidays) to 6 (drug discontinuation). We considered that patients had good adherence behaviors for ART if they reported no drug omissions or drug holidays for any of their drugs; moderate adherence if they reported some drug omissions or short drug holidays for at least one of their drugs; and poor adherence if they reported long drug holidays (>6 days), systematically skipped a daily dose, or discontinued at least one of their drugs.

Participants’ self-reported data were also enriched by data extracted from their medical records (age, sex, date of diagnosis, last CD4 count, current antiretroviral treatment, etc.) and from the MOTUHS study (educational level, individual income, and HIV-1 RNA viral load; virological failure was considered with HIV-1 RNA viral load >100 copies/mL [26]).

### Analysis

#### Description of PLWHIVs’ workload of care

We described the workload of care in our population by using five aggregate estimates: 1) total time spent in HRAs during the study period; 2) temporal dispersion of these HRAs (i.e., as the variance of the time intervals between HRAs); 3) ratio of health expenditures to patients’ revenue during the period; 4) opportunity cost, defined by the time patients could have spent doing something else; and 5) pill burden, defined as the total number of pills patients took every day.

However, our simple separate aggregate measurements may not accurately reflect the complexity of patients’ workload of care at the individual level. For example, two patients could have spent the same time in HRAs during the study period but with different activities, durations, monetary costs and opportunity costs. Therefore, we used an unsupervised learning method to uncover cohesive patterns of workload of care within our population. This method involved three steps. First, we computed a similarity distance between individuals by using Euclidean distances for the five key components of the workload of care described earlier (i.e., time spent in the different HRAs, temporal dispersion of HRAs, ratio of expenditures to patients’ revenue in the period, time patients could have spent in the different opportunities lost and total number of pills patients took every day). Second, we used individual difference scaling models to assess the structure of the resulting multidimensional data [27]. Finally, we identified homogenous groups of patients within this structure by using ascendant hierarchical clustering with Ward’s distance method.

#### Association between workload of care, adherence to ART and virological failure

We described how each workload of care pattern was associated with adherence to ART and virological status by using multilevel models, with study center as a random coefficient, and adjusted for age, sex, presence of multimorbidity (defined as the presence of at least one chronic condition not associated with HIV), square root transformed last CD4 count [28], time since start of ART (< 3 years vs >3 years) and educational level (primary school or less vs higher education).

#### Theoretical avoidable workload of care by re-organization of healthcare visits

We hypothesized that some of PLWHIVs’ workload of care was generated by the fragmentation of care and could be avoided if patients’ visits and tests could be grouped on the same days. We defined this theoretical “avoidable workload of care” as the resources invested by patients in terms of time, money and energy, which could be saved by grouping two doctor visits or tests: 1) occurring on different days; 2) spaced by less than 7 days; and 3) performed in the same geographic location (e.g., same hospital, same clinic, etc.). For each patient in our study, we assessed this theoretical avoidable workload of care in terms of gain in transportation time and money savings.

Analyses involved use of R v3.3 (http://www.R-project.org, the R Foundation for Statistical Computing, Vienna, Austria).

## Results

### Participants

From July 2016 to September 2016, 476 participants were included in the three study sites. Mean age was 44.8 years (SD = 9.3) and 345 (72%) participants were female. Participants were receiving ART for a mean of 7.2 years (SD = 3.6). One third of patients (n = 159, 33%) reported at least one other chronic condition not related to HIV, such as gastric ulcer or gastroesophageal reflux disease (n = 66); asthma (n = 27); hypertension (n = 27); or diabetes (n = 13) (Table 1). The sex ratio, duration of ART treatment and CD4 count at treatment initiation were globally similar for our patients and those from the IeDEA HIV registry in West Africa [29, 30]. In our sample, 347 (72.9%), 118 (24.8%) and 11 (2.3%) participants reported good, moderate and poor adherence behaviors for ART, respectively, and 65 (13.6%) had a viral load > 100 copies/mL and so considered with virological failure.

**Table 1:** Characteristics of people living with HIV infection (PLWHIV) in Abidjan, Côte d’Ivoire (n = 476). Total number of co-morbidities exceeds 100% because a patient may have multiple comorbidities.

Characteristics	Value	Missing data
**Age (years)–Mean (SD)**	44.8 (9.3)	-
**Female sex–No. (%)**	344 (72.3)	-
**Relationship status–No. (%)**		1 (0.2)
Single	162 (34.0)	
Married	241 (50.6)	
Widow, divorced, separated	72 (15.1)	
**Educational level–No. (%)**		-
Non-formal	96 (20.2)	
Primary	131 (27.5)	
Secondary	179 (37.6)	
Higher than secondary	70 (14.7)	
**Unemployed–No. (%)**	58 (12.2)	-
**Monthly individual income (USD)**		6 (1.2)
<99$	230 (48.3)	
>99$ and <166$	116 (24.3)	
>166$ and <498$	103 (21.6)	
>498$	21 (4.4)	
**Last CD4 count–Mean (SD)**	574 (273)	1 (0.2)
**Viral load <100 cp/mL–No. (%)**	65 (13.6)	-
**ART duration (years)–Mean (SD)**	7.2 (3.6)	-
**Total number of pills per day–Mean (SD)**	5.6 (3.9)	-
**Comorbidities–No. (%)**		-
Hypertension	27 (5.7)	
Dyslipidemia	2 (0.4)	
Chronic cardiac condition	8 (1.7)	
Stroke	3 (0.6)	
Chronic renal insufficiency	2 (0.4)	
Chronic pulmonary condition (besides asthma)	24 (5.0)	
Asthma	20 (4.2)	
Ulcer disease or gastro esophageal reflux disease	66 (14)	
Chronic liver condition (including infectious hepatitis)	15 (3.1)	
Diabetes	12 (2.5)	
Arthritis or osteoporosis	9 (1.9)	
Chronic neurologic condition	4 (0.8)	
Psychiatric condition	2 (0.4)	
Other	29 (6.1)	

### Description of PLWHIVs’ workload of care

#### Using aggregate estimates

**Fig 1:**
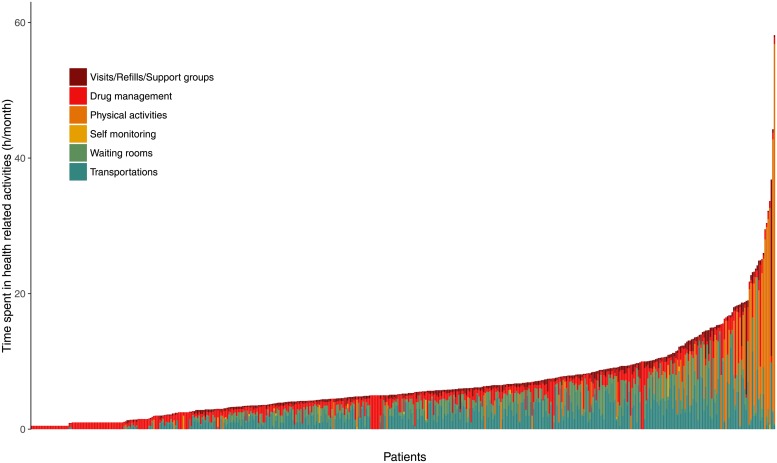
Time requirements for health-related activities (HRAs) for people living with HIV infection (PLWHIV) in Abidjan, Côte d’Ivoire (n = 476). Each vertical bar corresponds to a patient. The height of a bar represents the reported time spent performing HRA during the study period. Patients are ordered by increasing total time spent in HRA. Colors represent how this time is divided into the different kinds of HRAs.

Mean total time spent in HRAs was 6.7 hours (SD = 6.3). HRAs encompassed both “routine” at-home activities (e.g., medication management or spending extra time shopping to avoid eating specific foods) and non-routine out-of-home activities (e.g., doctor visits, pharmacy visits [including ART refills], tests, support groups) (S1 Table). Patients spent 49% of this time (SD = 32), on average, in transportation or waiting rooms (Fig 1).

Temporal dispersion of HRAs during the study period was uneven. The standard deviation of intervals between HRAs was 3.4 days (range 0–21 days), on average. Among patients with multiple HRAs (n = 305), these were grouped on same days for 139 (45%).

The ratio of health expenditures by patient revenue was 5% (SD = 11), on average. Patients spent a monthly mean expenditure in HRAs of 7.5 US dollars (SD = 15.2).

Opportunity costs involved patients performing HRAs instead of working (n = 496), doing housework (n = 197), relaxing (n = 88), caring for children (n = 47), spending time with friends (n = 41), or praying (n = 17).

Concerning pill burden, patients reported taking 5.6 pills per day (SD = 3.9), of which 2.8 (SD = 1.7) were related to ART.

Aggregate estimates for the workload of care of PLWHIV are presented in subgroups by type of clinic where patients received care (S2 Table) and by sex (S3 Table).

#### Uncovering cohesive patterns of workload of care within our population

**Fig 2:**
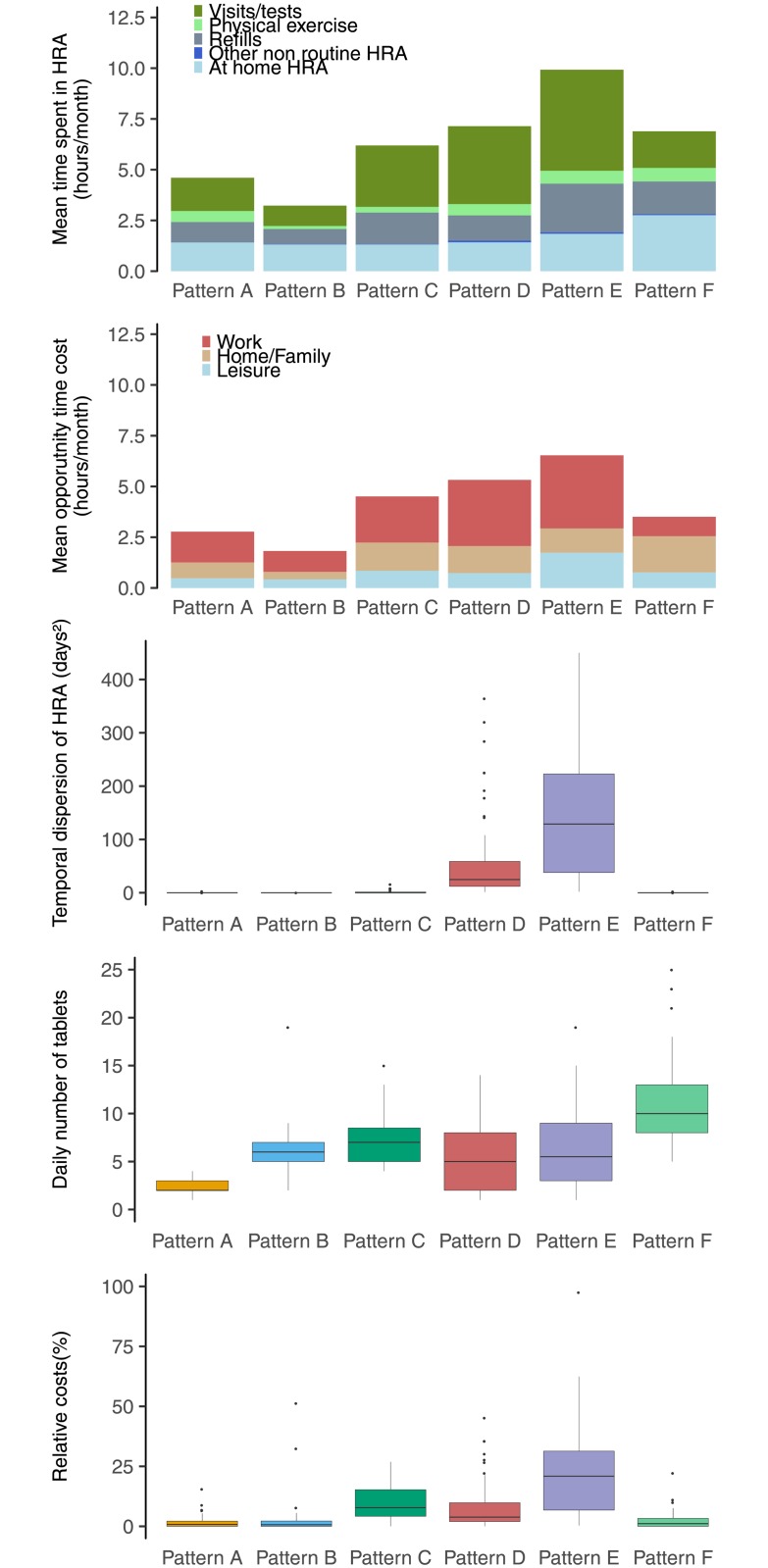
Workload of care in each pattern of workload of care identified (n = 476). Each panel shows a different aspect of the workload of care (time spent in the different HRAs, opportunity costs, daily number of pills taken, temporal dispersion of health activities and ratio of health expenditures to patients’ revenue). Patients are grouped according to the homogeneous patterns of workload of care identified by unsupervised learning methods (named A, B, C, D, E and F).

Our data exhibited high heterogeneity in patients’ workload of care. For example, patients’ investment of time in healthcare could vary from half an hour per month (for patients reporting spending approximately 1 minute per day in drug taking) to 58 hours per month (for patients with multiple visits and following advice to spend a regular time doing physical activity). Our unsupervised learning method identified six cohesive patterns of workload of care in our population. All patterns displayed a comparable mixture of activities and opportunity costs. However, they differed in mean total time spent in HRAs, temporal dispersion of HRAs, pill burden, and health expenditures. Pattern A involved patients with a “low workload of care”, taking few pills every day (mean 2.2 [SD = 0.8]), with a low temporal dispersion of HRAs (mean 0.07 [SD = 0.36]) and spending few of their revenues in healthcare (mean 1% [SD = 2]). Patterns B, C and D involved patients with a “moderate workload of care,” who took more pills every day (from 5.5 [SD = 3.6] to 7.2 [SD = 2.6]), with low to moderate temporal dispersion of HRAs and with low to moderate health expenditures (from 3% to 10% of their revenues, on average). Pattern E involved patients with a “high workload of care,” who had a high temporal dispersion of HRAs and high health expenditures (26% of their revenues, on average). Pattern F involved patients with a “high pill burden” (mean number of drugs taken every day 11 [SD = 4.5]) (Table 2 and Fig 2).

**Table 2:** Characteristics of PLWHIV in each workload of care pattern identified (n = 476).

	Pattern A “Low workload of care” (n = 134)	Pattern B “Moderate workload of care” (n = 114)	Pattern C “Moderate workload of care” (n = 47)	Pattern D “Moderate workload of care” (n = 86)	Pattern E “High workload of care” (n = 42)	Pattern F “High pill burden” (n = 53)
**Monthly time spent in HRA (minutes)–Mean (SD)**	362 (407)	197 (128)	378 (152)	467 (264)	739 (356)	614 (602)
**Monthly number of HRA–Mean (SD)**	1.4 (1.6)	1.1 (1.1)	2.5 (0.9)	3.2 (0.9)	3.6 (1.4)	1.4 (1.3)
**Number of medications taken everyday–Mean (SD)**	2.2 (0.8)	6.0 (1.8)	7.2 (2.6)	5.5 (3.6)	6.5 (4.4)	11 (4.5)
**Time dispersion of HRA within the month (variance of the interval between two visits)–Mean (SD)**	0.07 (0.36)	0 (0.03)	1.7 (3.2)	52.3 (70.6)	148 (126.5)	0.1 (0.4)
**Number (%) of patients performing physical exercise**	20 (15)	8 (7)	2 (4)	9 (10)	5 (12)	10 (19)
**Number (%) of patients with a work opportunity cost**	54 (40)	39 (34)	26 (55)	54 (63)	27 (64)	13 (25)
**Number (%) of patients with a housework opportunity cost**	26 (19	16 (14)	16 (34)	24 (28)	9 (21)	17 (32)
**Relative financial costs (US dollars)–Mean (SD)**	1(2)	3 (12)	10 (8)	8 (9)	26 (24)	2 (4)
**Age (years)–Mean (SD)**	44.3 (8.2)	45.7 (9.2)	47 (9.5)	43.9 (10.8)	44.4 (9.1)	44.2 (9.2)
**Number (%) of multimorbid patients**	46 (34)	32 (28)	26 (55)	38 (44)	14 (33)	19 (36)
**Duration under ART (years)–Mean (SD)**	6.4 (3.6)	7.1 (3.4)	8.3 (3.6)	7.3 (3.8)	7.3 (3.6)	8.0 (3.5)
**Number (%) of patients with imperfect adherence**	38 (28)	36 (32)	10 (21)	23 (27)	11 (26)	11 (21)
**Number (%) of patients in virological failure**	16 (12)	11 (10)	6 (13)	11 (13)	6 (14)	15 (28)

### Association between workload of care, adherence to ART and virological failure

No pattern of workload of care was associated with adherence to ART (S4 Table). However, virological control was significantly better for patients with a Pattern A workload of care (low workload) than other patterns (S5 Table): OR = 0.32 [95% CI 0.15–0.69] (p = 0.003) (S5 Table).

### Theoretical avoidable workload of care

**Fig 3:**
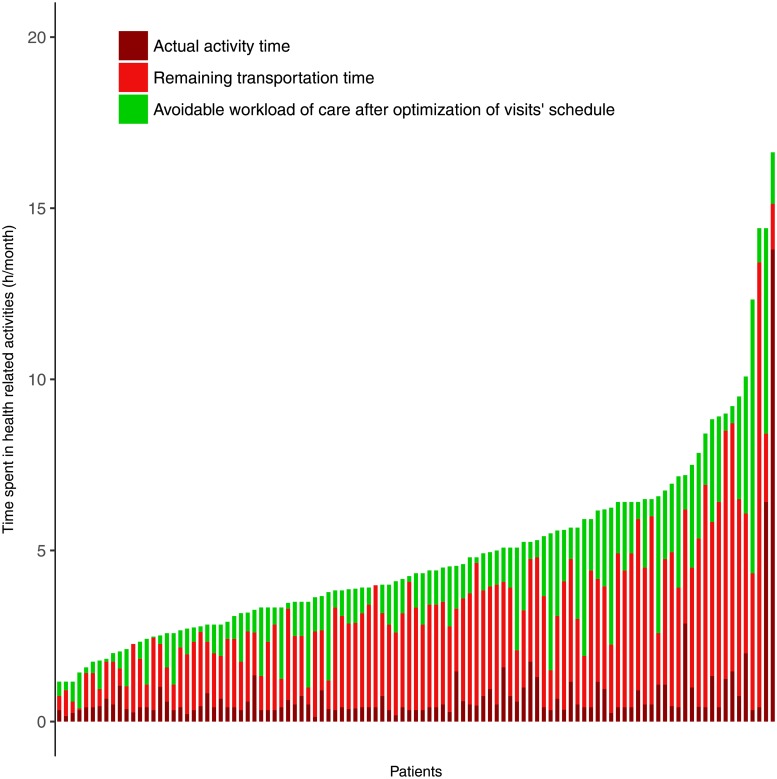
Avoidable workload of care by optimizing visit schedules to minimize transportation times (n = 107). Each bar corresponds to a patient. Only patients with ≥ 2 HRAs during the study period that could be theoretically grouped on the same day are shown. The height of a bar represents the reported time spent performing out-of-home HRAs during the study period (we did not represent the time spent doing routine HRAs such as medication management). The green portion of the bar indicates the avoidable workload of care resulting from grouping visits on the same days.

In our sample, 107 PLWHIV (22% of the total population and 35% of the patients with ≥2 non-routine HRAs) had at least two clinic visits or tests that could have been theoretically grouped to minimize transportation time. For these patients, mean gain of transportation time by reorganization of non-routine HRAs would be 84 min (SD = 77 min) per month, representing a mean decrease of 21% of their time devoted to non-routine HRAs (Fig 3). Potential money savings for these patients was 2.9 US dollars (SD = 4.1) per month, on average, representing a mean decrease of 19% in reported health expenditures within the study period.

## Discussion

To our knowledge, this is the first study to comprehensively describe the workload of care of PLWHIV in Sub-Saharan Africa in terms of time spent in health activities, opportunity and financial costs. Two main results emerged from our study. First, the workload of care for “chronic” PLWHIV (i.e., those under ART for at least 1 year) is complex, with high inter-individual heterogeneity. Our analysis led to the identification of six different patterns in the workload of care of PLWHIV in terms of number of, time spent in and frequency of HRAs; pill burden; or health expenditures. After adjustment for confounders, patients in the “low workload of care pattern” were more frequently virologically controlled. This association may support theoretical models hypothesizing that higher workload of care could lead to disengagement from care by PLWHIV [12]. Second, we showed that simple adjustments in the schedule of patients’ visits could reduce both time spent in health activities and transportation expenditures by approximately 20% for patients with multiple activities during the month.

In the literature on chronic illnesses, the workload of care has usually been summarized by simple estimates such as the total time spent in HRAs or health expenditures [31, 32]. However, such measures are simplistic and ignore the complexity of the workload of care. Two patients could have spent the same amount of time in HRAs but with a complete different time allocation and different opportunity costs. By combining the Daily Reconstruction Method, a method inspired from sociology, and advanced statistical methods, we were able to microscopically describe and identify emerging patterns of the workload of care in our population. We highlight that key indicators to discern patients’ workload of care were pill burden, health expenditures and temporal dispersion of health activities. Pill burden is well known to be an important factor of patients’ burden of treatment and to affect virological suppression of PLWHIV [33]. Single-tablet regimens have been found useful to reduce the number of ART pills taken by patients and improve their outcomes [34], but our study showed that 73% of our patients were still under multiple-tablet ART regimens (mean number of ART pills taken daily: 2.8 [SD = 1.7]). In our study, PLWHIV had high health expenditures even though ART and CD4 count tests are free of charge in Côte d’Ivoire [21, 35]. Indeed, besides these medications, patients still need to pay for other medications and other biological tests, visits, and transportation. In a context of resource scarcity, these costs may play an even greater role in patients’ disengagement from HIV care [36]. Finally, temporal dispersion of health activities and especially frequent visits and refills to obtain treatment have been identified as contributing to the burden of treatment for PLWHIV [37] and for patients with chronic conditions in general [6]. Some trials have started investigating how re-organization of care services in Sub-Saharan Africa could reduce the workload of PLWHIV [38, 39]. Regardless, much work still needs to be done. In this study, we showed that simple adjustments in visit schedules could theoretically reduce the number of days lost to non-routine HRAs and health expenditures for patients with multiple visits. In the present study, we explored only the gain from grouping visits and tests on the same days.

Our study is one of the first to quantify the impact of workload of care variables (time required per visit, frequency of visits, etc.) on patients’ adherence to ART and virological failure in Sub-Saharan Africa. During analysis, we showed an association between high workload of care and virological failure. Due to the cross-sectional nature of our study, our results are not sufficient to conclude on the existence of a causal link between workload of care and engagement in HIV care. These results still support qualitative studies and models on the factors associated with the HIV care continuum in low- and middle-income countries [12, 40, 41]: avoidable workload of care may contribute to poor engagement in the HIV continuum, non-adherence to treatment and worse outcomes [11, 12]. Prospective research is needed to evaluate the impact of simple and low-cost adjustments in visit schedules to reduce patients’ workload of care on “hard” outcomes such as adherence and disease control.

Our study has some limitations. Workload of care estimates from this study must be generalized with caution. Indeed, despite recruiting consecutive participants, with a population structure close to those described in large epidemiological studies in West Africa [29, 30], participants in our study were all located in a single large metropolitan city in Sub-Saharan Africa. Similarly, health expenditures may be country- and region-specific. Second, we may have underestimated the workload of care because interviews were focused on “activities” (i.e., “doing” the work, monitoring and appraisal) while not asking about the time and energy spent in making sense of health information, planning care or enrolling informal caregivers [42]. Third, we used an existing patient-reported instrument to assess adherence behaviors for ART [25]. This scale was not adapted specifically for our context, but its simplicity (e.g., use of icons and graphics to help respondents) limited the risk of bias due to cultural differences. Finally, when estimating the theoretical avoidable workload of care, we did not take into account the capacity of facilities to increase the number of patients using each health service (physicians, tests, ART refills), especially in the current context in which the number of patients under ART is booming.

Our findings have several implications. Although most of our patients (85%) had achieved viral suppression, patients still had a heavy workload of care in terms of time, opportunity and financial costs. Thus, clinicians, researchers and decision makers must start taking into account the workload of care and burden of treatment for PLWHIV to achieve the “fourth 90”, that is, “good health-related quality of life for all patients who achieve viral suppression” [43]. Furthermore, our results point to the need to challenge the current organization of HIV care in Sub-Saharan Africa. In our study, half of the time devoted to HRAs was spent in transportation and waiting rooms. Simple adjustments to visit schedules could significantly reduce the workload of care for these patients and thus minimize the disruption healthcare has in their lives.

## Conclusion

Our study shows that PLWHIV deal with complex care involving multiple different time-consuming health-related activities. This workload of care could be avoided in part by simple adjustments in visit schedules for patients and could help improve retention in care and quality of life for these patients.

## Supporting information

S1 Table Characteristics of health-related activities reported by people living with HIV infection (PLWHIV). (DOCX)

S2 Table PLWHIVs’ workload of care by type of clinic they consulted. (DOCX)

S3 Table PLWHIVs’ workload of care by sex. (DOCX)

S4 Table Results from the multilevel model assessing the association between the patterns of workload of care of PLWHIV and adherence to ART. (DOCX)

S5 Table Results from the multilevel model assessing the association between the patterns of workload of care of PLWHIV and virological control. (DOCX)
