# Supplementary material for: Avoidable workload of care for patients living with HIV infection in Abidjan, Côte d’Ivoire: A cross-sectional study
Source: PLoS One. 2018 Aug 24;13(8):e0202911. doi: 10.1371/journal.pone.0202911 (PMC6108500; doi:10.1371/journal.pone.0202911)
Supplement: S1 Table — (DOCX) [file pone.0202911.s001.docx]

**S1 Table. Characteristics of health-related activities (HRAs) reported by people living with HIV infection (PLWHIV)**.

| **HRAs** | **Number (%) of patients who performed the activity in the month** | **Number (%) of activities difficult to integrate in patients’ daily lives** | **Actual activity time (min) -**  **Mean (SD)** | **Transportation time (min) -**  **Mean (SD)** | **Waiting time (min) -**  **Mean (SD)** | **Out of pocket Costs (US dollars)**  **Mean (SD)** |
| --- | --- | --- | --- | --- | --- | --- |
| **Routine HRAs performed at home** | | | | | | |
| Manage medications (n=476) | 476 (100.0%) | 97 (20.4 %) | 87 (95) | - | - | - |
| Physical exercise (n=54) | 54 (11.3 %) | 2 (3.7 %) | 641 (652) | - | - | - |
| Dietary changes (n=89) | 89 (18.7 %) | 21 (23.6 %) | - | - | - | - |
| **Non-routine HRAs reported by participants (n=944)** | | | | | | |
| Organize appointments (n=2) | 2 (0.4 %) | 1 (50.0 %) | 4 (1) | 30 (42) | 1 (2) | 1.3 (1.1) |
| Visits to healthcare providers (n=323) | 275 (57.7 %) | 82 (25.4 %) | 21 (45) | 66 (66) | 53 (57) | 4.8 (11.3) |
| Exams and tests (n=188) | 183 (38.4%) | 61 (32.4 %) | 6 (6) | 88 (92) | 41 (43) | 4.0 (9.3) |
| Visits to traditional practitioners (n=15) | 15 (3.1 %) | 2 (13.3 %) | 22 (25) | 43 (44) | 3 (4) | 3.9 (6.8) |
| Pharmacy and refills (n=374) | 323 (67.8 %) | 81 (21.7 %) | 9 (12) | 57 (55) | 30 (62) | 3.1 (4.7) |
| Organize drugs at home (n=8) | 8 (1.7 %) | 2 (25.0 %) | 15 (14) | - | - | - |
| Health-related shopping (n=1) | 1 (0.2 %) | 0 (0.0 %) | 30 | - | - | - |
| Support or educational groups (n=22) | 17 (3.6 %) | 5 (22.7 %) | 125 (107) | 141 (79) | 40 (69) | 1.8 (1.0) |
| Health-related administrative tasks (n=6) | 6 (1.3 %) | 0 (0.0%) | 7 (7) | 40 (47) | 42 (57) | 1.1 (1.3) |
| Other (n=5) | 5 (1.0 %) | 0 (0.0%) | 45 (56) | 33 (52) | 0 | 0.3 (0.4) |
