# Supplementary material for: Avoidable workload of care for patients living with HIV infection in Abidjan, Côte d’Ivoire: A cross-sectional study
Source: PLoS One. 2018 Aug 24;13(8):e0202911. doi: 10.1371/journal.pone.0202911 (PMC6108500; doi:10.1371/journal.pone.0202911)
Supplement: S2 Table — (DOCX) [file pone.0202911.s002.docx]

**S2 Table. PLWHIVs’ workload of care by type of clinics attended**

| **Workload of care estimate** | **Attended public HIV clinics**  **(n=284)** | **Attended private HIV clinics**  **(n=192)** |
| --- | --- | --- |
| Number of HRAs/month – Mean (SD) | 2.1 (1.4) | 1.8 (1.5) |
| Total time spent in HRAs (hours/month) – Mean (SD) | 7.2 (6.1) | 6.0 (6.5) |
| Temporal dispersion of the HRAs  (as the variance between two HRAs, in days²) – Mean (SD) | 31 (76) | 10 (39) |
| Health expenditures (US dollars) – Mean (SD) | 9.3 (18) | 4.7 (9) |
| Ratio of health expenditures to patients’ revenue – Mean (SD) | 7.8 (14.9) | 3.3 (6.2) |
| Total number of pills patients took every day – Mean (SD) | 5.7 (4.2) | 5.4 (3.4) |
