# Supplementary material for: Avoidable workload of care for patients living with HIV infection in Abidjan, Côte d’Ivoire: A cross-sectional study
Source: PLoS One. 2018 Aug 24;13(8):e0202911. doi: 10.1371/journal.pone.0202911 (PMC6108500; doi:10.1371/journal.pone.0202911)
Supplement: S3 Table — (DOCX) [file pone.0202911.s003.docx]

**S3 Table. PLWHIVs’ workload of care by sex**

| **Workload of care estimate** | **Men**  **(n=132)** | **Women**  **(n=344)** |
| --- | --- | --- |
| Number of HRAs/month – Mean (SD) | 2.2 (1.4) | 1.9 (1.4) |
| Total time spent in HRAs (hours/month) – Mean (SD) | 7.6 (7.1) | 6.4 (5.9) |
| Temporal dispersion of the HRAs  (as the variance between two HRAs, in days²) – Mean (SD) | 32 (74.5) | 19 (60) |
| Health expenditures (US dollars) – Mean (SD) | 8.7 (16.7) | 7.0 (14.5) |
| Ratio of health expenditures to patients’ revenue – Mean (SD) | 4.5 (10) | 6.6 (13) |
| Total number of pills patients took every day – Mean (SD) | 5.6 (4.0) | 5.6 (3.8) |
