# Supplementary material for: Avoidable workload of care for patients living with HIV infection in Abidjan, Côte d’Ivoire: A cross-sectional study
Source: PLoS One. 2018 Aug 24;13(8):e0202911. doi: 10.1371/journal.pone.0202911 (PMC6108500; doi:10.1371/journal.pone.0202911)
Supplement: S4 Table — (DOCX) [file pone.0202911.s004.docx]

**S4 table. Results from the multilevel model assessing the association between the patterns of PLWHIVs’ workload of care and adherence to ART.** The multilevel model used study center as a random coefficient, and was adjusted for age, sex, presence of multimorbidity (defined as the presence of at least one chronic condition not associated with HIV), square root transformed last CD4 count, time since start of ART (< 3 years vs >3 years) and educational level (primary school or less vs higher education). Odds ratio (OR) > 1 indicates greater odds of reporting high adherence to ART.

| **Variable included in the model** | **OR [95% CI]** | **p** |
| --- | --- | --- |
| Age | 1.04 [1.02–1.07] | 0.0009 |
| Female sex | 1.02 [0.61–1.69] | 0.95 |
| Presence of multimorbidity | 1.01 [0.65–1.58] | 0.95 |
| CD4 count (square root) | 1.03 [0.99–1.06] | 0.17 |
| Duration under ART (>3 years) | 1.72 [0.98–3.02] | 0.05 |
| Education (higher education) | 0.70 [0.45–1.07] | 0.10 |
| Workload of care pattern B (vs A) | 0.65 [0.30–1.42] | 0.27 |
| Workload of care pattern C (vs A) | 0.52 [0.23–1.15] | 0.10 |
| Workload of care pattern D (vs A) | 0.77 [0.29–2.05] | 0.60 |
| Workload of care pattern E (vs A) | 0.78 [0.33–1.81] | 0.55 |
| Workload of care pattern F (vs A) | 0.86 [0.32–2.32] | 0.76 |
| Higher workload of care (pattern B-F) (vs A) | 0.66 [0.32-1.35] | 0.26 |
