# Supplementary material for: Avoidable workload of care for patients living with HIV infection in Abidjan, Côte d’Ivoire: A cross-sectional study
Source: PLoS One. 2018 Aug 24;13(8):e0202911. doi: 10.1371/journal.pone.0202911 (PMC6108500; doi:10.1371/journal.pone.0202911)
Supplement: S5 Table — (DOCX) [file pone.0202911.s005.docx]

**S5 table. Results from the multilevel model assessing the association between the patterns of PLWHIVs’ workload of care and virological control.** The multilevel model used study center as a random coefficient and was adjusted for age, sex, presence of multimorbidity (defined as the presence of at least one chronic condition not associated with HIV), square root-transformed last CD4 count, time since start of ART (< 3 years vs >3 years), adherence to ART and educational level (primary school or less vs higher education). Odds ratio (OR) < one indicates lower odds of being in virological control.

| **Variable included in the model** | **OR** | **p** |
| --- | --- | --- |
| Age | 1.0 [0.97–1.03] | 0.97 |
| Female sex | 1.09 [0.54–2.19] | 0.81 |
| Presence of multimorbidity | 1.18 [0.65–2.15] | 0.59 |
| CD4 count (square root) | 0.86 [0.82–0.91] | <0.0001* |
| Duration under ART (>3 years) | 3.29 [1.08–10.05] | 0.03* |
| Education (higher education) | 0.67[ 0.36–1.23] | 0.19 |
| Adherence to ART | 0.42 [0.22–0.78] | 0.006* |
| Workload of care pattern B (vs A) | 0.37 [0.15–0.89] | 0.02* |
| Workload of care pattern C (vs A) | 0.27 [0.10–0.70] | 0.006* |
| Workload of care pattern D (vs A) | 0.34 [0.10–1.13] | 0.07 |
| Workload of care pattern E (vs A) | 0.37 [0.14–0.98] | 0.04* |
| Workload of care pattern F (vs A) | 0.26 [0.08–0.83] | 0.006* |
| Higher workload of care (pattern B-F) (vs A) | 0.32 [0.15-0.69] | 0.003 |
